# How health seeking behavior develops in patients with type 2 diabetes: a qualitative study based on health belief model in China

**DOI:** 10.3389/fpubh.2024.1414903

**Published:** 2024-07-09

**Authors:** Qiu-hui Du, Zi-chen Zhang, You Yang, Xiao-xi Luo, Li Liu, Hong-hong Jia

**Affiliations:** ^1^Department of Nursing, Harbin Medical University (Daqing), Daqing, China; ^2^Department of Ultrasound, Affiliated Hospital of North Sichuan Medical College, Nanchong, China

**Keywords:** type 2 diabetes mellitus (T2MD), health seeking behavior, health belief model, qualitative design, China

## Abstract

**Background:**

Type 2 diabetes(T2DM) is a global health problem which is accompanied with multi-systemic complications, and associated with long-term health burden and economic burden. Effective health seeking behavior (HSB) refers to reasonably utilize health resources, effectively prevent and treat diseases, and maintain health. Effective health seeking behavior (HSB) is vital to mitigate the risk of T2DM complications. However, health seeking behavior for T2DM patients remains sub-optimal worldwide.

**Objective:**

The study aimed to explore the internal logic of how health seeking behavior of T2DM patients develops and the influencing factors of health seeking behavior. With a view to provide a reference basis for improving the health seeking behavior situation of T2DM patients.

**Methods:**

This study was conducted at an integrated tertiary hospital in China. People who were diagnosed with T2DM, capable of expressing clearly and had no mental illness, were approached based on a purposive sampling. The experience of T2DM and health seeking behavior were collected via in-depth interviews. A theory-driven thematic analysis based on *Health Belief Model (HBM)* was applied for data analysis. Inductive reasoning was used to identify emerging themes which were not included in HBM.

**Results:**

26 patients with T2DM were included in the current study. Seven themes were identified, including: (1) T2DM diagnosis and severity; (2) T2DM treatment and management; (3) Perceived susceptibility of diabetes progression; (4) Perceived severity of diabetes progression; (5) Perceived benefits of health seeking behavior; (6) Perceived barriers of health seeking behavior; (7) Perception of behavioral cues. Generally, patients with T2DM lacked reliable sources of information, considered T2DM to be slow-progressing and without posing an immediate threat to life. Consequently, they did not fully grasp the long-term risks associated with T2DM or the protective effects of health seeking behavior.

**Conclusion:**

This study highlighted the challenges in health seeking behavior for patients with T2DM. It suggested that future interventions and strategies should involve multi-faceted approaches, targeting healthcare providers (HCPs), patients with T2DM, and their support networks. This comprehensive strategy can help patients better understand their condition and the importance of effective health seeking behavior. Ultimately, enhancing their capacity for adopting appropriate health-seeking practices.

## Introduction

Type 2 diabetes Mellitus (T2DM) is the most prevalent form of diabetes, accounting for 90–95% of all diabetic cases, and it poses a significant global health and socioeconomic challenge (1). The global prevalence of diabetes in adults (aged 20–79) was estimated to be 10.5% in 2021, and this figure is projected to rise to 12.2% by 2045 (2). As a result, the global health expenditures related to diabetes were estimated to be 966 billion USD in 2021 and are expected to reach 1,054 billion USD by 2045 (2). The high prevalence and rapid increase in diabetes make it one of the most critical public health challenges of the 21st century. The World Health Organization (WHO) has identified diabetes as one of the five priority noncommunicable diseases in its action plan to address the health challenges (3).

Globally, 240 million people estimated to be undiagnosed. And in China, approximately 50.5% of the adults (aged 20–79) with diabetes remaining undiagnosed (4). According to epidemiological surveys in China, the treatment rate of diabetes is only 32.2% (5). This lack of diagnosis, coupled with a low treatment rate, promotes the development of diabetes complications, which can be disabled and even fatal to those affected.

Research in 2023 by Liu et al. (6) revealed that over two-thirds of T2DM patients in China had at least one complication, and the percentage of patients with three or more complications had risen from 12% in 2014 to 50% in 2019. Diabetes complications, such as nontraumatic lower limb amputation and blindness have severely impacted the quality of life for T2DM patients, leading to disability. These complications have also been identified as the primary cause of death among diabetic patients (7). Moreover, patients with complications from diabetes incur significantly higher health seeking expenses, approximately 3.36 times higher than those without complications (8).

The insidious and progressive nature of diabetes highlights the importance of early detection, prompt diagnosis and ongoing care with regular evaluation to mitigate the growing burden of this condition (7). While there is strong evidence that access to good care, education and medication can control diabetes and its complications, it is essential to understand the health seeking behavior of T2DM patients, as it plays a crucial role in determining whether they receive appropriate care and treatment. This understanding can shed light on the existing under-utilization of healthcare services despite increased access (9).

Previous research has found that health seeking behavior in diabetes is complex, influenced by various factors including economic and cultural (10, 11), cognitive (12, 13), disease attitude (14), health seeking attitude (15), self-efficacy (16), healthcare system factors (17), social support (18) and so on. However, previous studies always adopted quantitative research methods to explore the influencing factors of diabetes patients’ health seeking behavior. Few studies adopted qualitative research methods to deeply understand the formation and experience of diabetes patients’ health seeking behavior. Qualitative research adopted open questions allowed us to fully consider the influence of social psychological factors on behavior, to understand more potential problems and information, better understand how health seeking behavior develops in patients with type 2 diabetes, so as to draw more accurate, full and comprehensive conclusions.

Health belief model (HBM) was proposed by American psychologists Hochbaum and others, that application of social psychology methods to explore the formation mechanism and influencing factors of behavior from the perspective of people’s health beliefs is an important theoretical model for explaining people’s health behavior (19). It has been widely applied to explain and predict individual health behaviors related to disease prevention and management (20), that is why making it a valuable framework to examine why patients with T2DM seek or do not seek health services (21). In this study, a qualitative design was adopted to gather the experiences of T2DM patients from the time of diagnosis to the formation of stable health seeking behavior. The objective was to explore the internal logic of how health seeking behavior of T2DM patients develops and the influencing factors of health seeking behavior based on HBM. With a view to provide a reference basis for improving the health seeking behavior situation of T2DM patients.

## Methods

### Study design

This qualitative descriptive study utilizes semi-structured in-depth interviews to collect the experiences, perspectives, and attitudes of the research subjects in seeking healthy behaviors through face-to-face semi-structured conversations, as well as to gain a deeper understanding of their underlying thoughts and motivations. Record the entire interview process and transcribe the interview data within 24 h after the interview ends. The subsequent data analysis is based on thematic analysis of the interview content and development of the conceptual framework. The study design and reporting followed the Qualitative Research Comprehensive Reporting Standard (COREQ) checklist.

### Settings

The research was conducted in the Endocrine Department of a Hospital in Daqing, China. The Fourth Hospital of Daqing is a national tertiary comprehensive hospital that offers a wide range of medical services and has an annual outpatient volume of nearly 0.5 million people. As a comprehensive hospital, patients seeking health at this hospital have various sociodemographic backgrounds and experiences in seeking health.

### Participants

According to “the American diabetes association diabetes diagnostic criteria (2023),” and any result higher than the standard was diagnosed as T2DM. Eligible criteria: (1) Diagnosed as type 2 diabetes (Diagnosed by a physician); (2) Age ≥ 18 years old; (3) Conscious, with stable vital signs, and the ability to communicate normally; (4) Willingness to participate and able to provide informed consent. Exclusion criteria: (1) Patients with cognitive impairment; (2) Patients with mental disorders. (3) Patients with gestational diabetes. Purposive sampling and maximum variation sampling strategies (including occupation and disease duration) was adopted to ensure the inclusion of T2DM patients with diverse characteristics.

### Interview guideline

A semi-structured interview guideline was developed and pilot-tested to collect data based on health-seeking behavior. The guideline contained open-ended questions focused on participants’ experiences, beliefs regarding diabetes, and how they formed their current health-seeking behavior. According to the HBM (22), individual adoption of a health behavior depends on their belief in its effectiveness and their desire to avoid potential disease progression.

**Figure 1:**
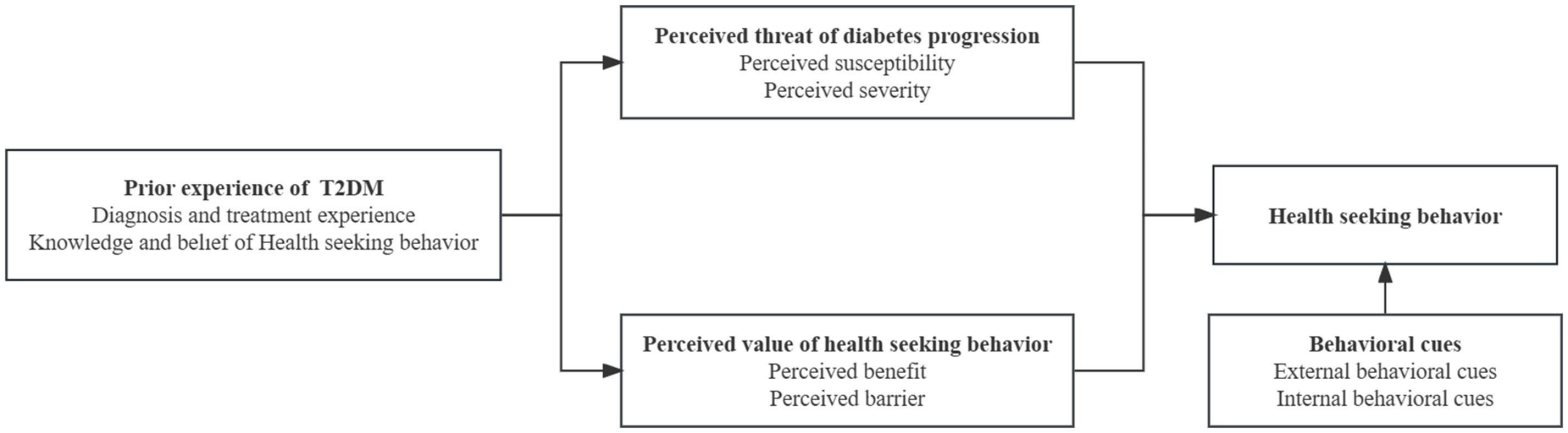
Framework of interview guideline based on HBM.

According to HBM (Figure 1), whether the patients with T2DM developed standardized HSB depends mainly on their perceived value of HSB and perceived threat of potential diabetes progression consequences. Among them, perceived value is a systematic assessment of the benefits and obstacles of HSB, and perceived threat is an overall assessment of the susceptibility and severity of the consequences of potential diabetes progression. In addition, the experience of T2DM patients has affected and shaped the HSB of diabetic patients. Based on the above questions, an interview guideline for this study was developed (Table 1). Due to the high prevalence of diabetes in the world, behavioral norms may affect the HSB of diabetic patients. Therefore, we also collected the demographic data of participants.

**Table 1:** Interview questions about T2DM patients based on HBM.

Topic one: Experience of T2DM
When and how were you diagnosed with diabetes?
Please tell me about your experience of diabetes?
Topic Two: Perceived threat of diabetes progression consequence
What do you think about the possibility of diabetes progression? Why?
What do you think about the progression of diabetes?
Topic Three: Perceived value of health seeking behavior
What do you know about the benefit of health seeking behavior?
Is any barrier of health seeking behavior? / Why you do not want to seeking health service?
Topic Four: Behavior cues
Are you influenced by external factors when you seeking health service?
Are you influenced by Internal factors when you seeking health service?

### Data collection

Nurses at the Endocrinology Department of Daqing Fourth Hospital identified potential participants, informed them about the study, and invited them to participate. The interviews were conducted by a pair of authors from July to August 2023 and had a duration of 25 to 48 min each. The interview adopts an open-ended questioning method, during which the interviewer carefully listens, observes and records their facial expressions, emotional changes, and body language, and encourages them to express their true feelings. Flexibly adjust the interview procedure and content based on the specific situation of the interview. All interviews were conducted face-to-face and recorded using digital audio recording devices. The interviewers were then transcribed verbatim by two researchers at the same time. Data saturation was reached after 23 interviews, and an additional three interviews found that the explanatory dimensions of each encoding or topic began to repeat, and no other explanations were found for the encoding or topic, that confirmed data saturation. In total, 26 interviews were completed.

### Data analysis

The data analysis adopts a theoretical driven topic analysis method based on HBM, which includes six steps: data familiarization, generating initial encoding, searching for topics, reviewing topics, defining and naming topics, and writing a manuscript. Two researchers independently and repeatedly read the text, identifying meaningful unites and categorizing keywords and phrases, and used an iterative approach whereby codes were continuously revised and refined throughout the analysis. Any discrepancies during the encoding process were solved by joint discussed with another researcher to ensure that both broader level and emergent sub-themes were exhaustively captured. After the coding process, one researcher used NVIVO Version 12.5.0 software to assist in grouping the codes into themes. These themes were categorized based on the initial coding under the umbrella of health-seeking behavior. The sub-themes that appeared were driven by classification data based on the initial codes within the same main theme. Finally, review all identified themes and sub-themes based on the initial encoded and transcribed text. To ensure credibility, the results were fed back to the participants for further confirmation.

### Rigor

This study conducted analysis under the guidance of the Health Belief Model (HBM), ensuring the scientific and objective nature of the study. The data analysis was conducted independently by two researchers, any discrepancies during the encoding process were solved by joint discussed with another researcher to avoid the authors’ subjective. We also have adopted an iterative approach in data analysis, which helps us gain a deeper and more accurate understanding of the data and to some extent avoids researcher bias. The research team of this study includes three researchers with associate professor or above titles who have rich qualitative research experience, and three graduate students who have received systematic qualitative research training, different roles and backgrounds have also contributed to avoiding bias in research results. Finally, we invited three qualitative research experts review all identified themes and sub-themes based on the initial encoded and transcribed text to ensure that the whole text data is well presented and the relationships between themes were not distorted. In order to ensure credibility, the results were fed back to the participants for further confirmation, and they all expressed that the research results were consistent with their ideas.

## Results

A total of 26 patients diagnosed with T2DM were recruited for interviews. The demographic composition of the participants was as follows: 38% of the participants were female (*n* = 10); The age range of the participants was between 28 and 82 years; The education level of the participants varied from illiterate to holding a master’s degree; The duration of diabetes ranged from 2 year to 30 years, among them, over half of the participants (*n* = 14) had been living with T2DM for over 10 years, while 5 patients had been diagnosed with T2DM for less than 5 years; Participants came from diverse occupational backgrounds, including clerk, farmers, retirees, businessmen, civil servants, and others. Detailed information is presented in Table 2.

**Table 2:** Demographic data of the participant.

	Age (years)	Sex	Occupation	Education	Members of family	Complication	Confirmed time (years)
1	58	Female	Clerk	High School	Husband	Exist	15
2	47	Male	Teacher	Middle School	Wife	None	3
3	47	Male	Business man	Middle School	Wife	Exist	10
4	28	Male	Electrician	High School	Wife, daughter	None	2
5	59	Male	Clerk	Vocational school	Wife	Exist	8
6	60	Male	Retiree	Middle School	Wife, son’s family	Exist	20
7	52	Male	Functionary	Master	Wife, daughter	Exist	12
8	60	Male	Veterans	High School	Wife	Exist	7
9	51	Female	Labourer	High School	Husband	Exist	10
10	36	Male	Driver	Middle School	Wife, son	None	5
11	59	Male	Conductor	Associate	Wife	Exist	5
12	42	Male	Driver	Primary School	Wife	None	2
13	72	Male	Retiree	High School	Wife	Exist	28
14	63	Female	Retiree	Bachelor	Husband	Exist	10
15	55	Female	Business man	Middle School	Husband, son’s family	Exist	20
16	66	Female	Financial staff	Vocational school	Husband, son’s family	Exist	20
17	68	Female	Retiree	Associate	Son’s family	Exist	23
18	48	Male	Civil servant	Bachelor	Wife	Exist	20
19	52	Male	Business man	Bachelor	Wife	Exist	5
20	47	Female	Teacher	Associate	Husband	Exist	13
21	78	Female	Farmer	illiteracy	Husband	Exist	5
22	72	Female	Farmer	illiteracy	Husband	Exist	18
23	74	Male	Farmer	illiteracy	Live alone	Exist	5
24	68	Male	Farmer	Primary School	Wife	Exist	4
25	60	Male	Village cadre	Middle School	Wife	None	2
26	82	Female	Farmer	illiteracy	Son	Exist	30

### T2DM diagnosis and severity

*Question: “When and how were you diagnosed with diabetes? Please tell me about your experience of diabetes?”*

Most participants were diagnosed with diabetes when they experienced symptoms related to the condition or its complications. Small number of participants were diagnosed with diabetes when they performed physical examination. When informed of the results, most participants said they were not surprised because they had family history of diabetes and the physical symptoms also made them perceived that something’s wrong.

*When I went to the toilet, I found that the urine was sticky, when I stepped on the urine, it stuck to the sole of my shoe. I suspected that I had diabetes, because many people around me had diabetes, so I know it. Later, I checked the blood sugar and diagnosed it. (No. 2).*

However, in cases where there were no family history or apparent symptoms, participants often expressed surprise and disbelief regarding their diagnosis. They attributed the condition to irregular diet and rest.

*I was surprised at that time. I did not have a family history, and nothing is wrong, I could not believe it! Later, I went to other hospitals for examination, which also said that my blood sugar was high and diabetes was diagnosed. (No.13).*

Most participants believe that slightly elevated blood sugar levels that did not significantly disrupt their daily lives, and they perceived diabetes as a common and non-serious ailment. As a result, they did not initially manage their diabetes effectively or go for regular check-ups, as they considered it non-urgent.

*Diagnosed diabetes, but I did not pay attention to it, I did not take medicine, and did not check blood sugar. It was more than ten years later that I was hospitalized for the first time in 2009. I did all physical examinations, and the fasting blood sugar was about 12 then. (No.13).*

### T2DM treatment and management

Some participants initially managed their diabetes effectively through diet control and exercise. This early success sometimes led to overconfidence, causing participants to become less vigilant and stopped regular monitoring of blood sugar. It is worth noting that most participants said they never monitored postprandial blood glucose.

*At the beginning, I through exercise and diet to control my blood sugar, the effect was quite good at that time. Originally, I always monitored my fasting blood sugar. Then with confidence in blood sugar control, I occasionally monitored my fasting blood sugar, but I have never monitored my postprandial blood sugar. Nowadays, relying solely on diet and exercise cannot control blood sugar anymore. I always feel like I need to increase the dosage of hypoglycemic drugs after meals, otherwise I will not be able to control it. (No.14).*

Diabetes necessitates long-term disease management and control. While some participants could initially adhere to strict control measures, many eventually gave up on such rigorous management.

*When I first diagnosed diabetes, I went running at three o’clock every morning. I lost more than 15 kg and my blood sugar was well controlled. Later on, I thought it would be too hard to persist, so I gave up. (No.6).*

Participants experiences highlight the dynamic nature of T2DM, with individuals often experiencing fluctuations in their level of attention and vigilance toward the condition. When they observed that their blood sugar levels significantly elevated or worsened, they re-evaluated their disease and more active management and treatment. The onset of complications can serve as a powerful wake-up call, urging individuals to take their diabetes more seriously.

*I was so busy every day, so I did not pay attention to diabetes. Later, it became worse, and my blood sugar could not be controlled, so I had to go to the hospital to see a doctor. (No. 16).*

### Perceived threat of diabetes progression

#### Perceived susceptibility

*Question: “What do you think about the possibility of diabetes progression? Why?”*

When asked about the susceptibility of diabetes progression, some participants (*n* = 7) believed that their risk of diabetes complications was high. They based this belief on: family history, course of disease, age, personal physical condition, lifestyle and disease control.

*I think I may suffer from complications of diabetes in the future. I think that with the prolongation of the course of disease and the growth of age, the possibility of complications of the decline of physical function will also enhance. (No.5).*

A majority of participants (*n* = 16) believed that their risk of diabetes complications was low, there was currently no immediate threat of diabetes progression. Their reasoning included they belief that diabetes progression is a slow process; controlling blood sugar could prevent complications.

*My fasting blood sugar is generally not very high. Diabetes worsens slowly. When my blood sugar is high, I will feel my mouth is dry, at that time, I will monitor my blood sugar and take hypoglycemic drugs. At present, my disease is unlikely to progress. (No. 21).*

Three participants expressed that they never imagined the possibility of the progression of diabetes. Because they fear of considering it.

*I dare not think about the possibility of complications of diabetes. I am afraid that diabetes complications will drag my family down, so that, when I found that my blood sugar levels were very high, I feel uncertain and afraid. Thus, I have not measured blood sugar for a long time. (No. 6).*

#### Perceived severity

*Question: “What do you think about the progression of diabetes?”*

More than half of the participants (*n* = 16) considered the complications resulting from diabetes progression to be very serious. They expressed concerns about the impact on various aspects of their lives: life threat, organ damage, long-term consequences, psychological burden, possible need for insulin or hospitalization.

*I often experience hypoglycemia while sleeping at night, which makes me feel terrible. If I do not wake up during hypoglycemia, I may die. At that time, I wanted to be hospitalized. (No. 15).*

A group of participants (*n* = 10) felt that the symptoms they experienced were mild and did not impact their daily lives significantly, and they had less understanding of the severity of diabetes complications. They downplayed the seriousness of diabetes based on their current physical condition, and they were less alert to diabetes-related symptoms and potential complications.

*The progression of diabetes is not serious for me, I am old now. I feel numb in my hands and feet now, but I do not think it will have much impact on me, so I did not tell the doctor about this symptom when I went to the hospital for treatment, because it was less impact on my daily lives. (No. 21).*

### Perceived benefits and barriers of health seeking behavior

#### Perceived benefits

*Question: “What do you know about the benefit of health seeking behavior?”*

Participants had varied perceptions of the benefits of health seeking behavior, including it could reduce the threat of disease, could effectively control blood sugar and avoid (or delay) the occurrence of complications, could help diabetic patients understand their true health status, could increase their knowledge of diabetes, could reduce their and their families’ worries about their health status, it also can promote lifestyle changes and avoid further financial and care burdens on family members due to severe illness in the future.

*Health seeking behavior can delay the occurrence of complications, and I can absorb some experience in diabetes management. When blood sugar is well controlled, it also reduces the psychological burden on my family. (No.1).*

Some participants believed that diabetes was a condition that they could manage on their own, self-medication and lifestyle adjustments as sufficient and did not see the need for professional healthcare services. The idea that diabetes was incurable led some participants to question the purpose of health seeking behavior, they did not see how seeking healthcare could change their condition.

*I feel that diabetes is mainly controlled by myself. When I feel that the blood sugar is high, I will adjust the dosage of drugs. Because diabetes is incurable, there is no need to go to the hospital. (No. 25).*

#### Perceived barriers

*Question: “Is any barrier of health seeking behavior?*

When participants were asked about the barriers to health seeking behavior, several common obstacles emerged: the most frequently mentioned barrier was the financial burden of seeking healthcare. Many participants, particularly those in rural areas, cited concerns about spending too much money on medical expenses, the high self-payment expenses due to low insurance reimbursement rates were especially concerning.

*The New Rural Cooperative Medical Insurance we purchased has a reimbursement rate of 60% for hospitalization, which is not high and cannot be reimbursed for outpatient services. (No. 26).*

Participants noted that being too busy with work or family responsibilities was a significant barrier. They often felt they had no time to seek healthcare. Some participants believed that there was no difference between early and late health seeking behavior for a chronic condition like diabetes.

*Because I have children to take care of and I have to work, I usually do not go to the hospital. I only consider going to the hospital when it’s serious. (No. 4).*

Traffic barriers and cumbersome medical procedures were mentioned as obstacles, particularly by older adults and remote patients. Long travel times, complex registration and payment processes, and inconvenience in reaching healthcare facilities deterred them from seeking medical attention.

*I find it troublesome to go to the hospital. I need to take the bus for an hour, waiting in line to register and undergo a check-up. It will take me a whole day and make me very tired. (No. 9).*

*Question: “Why you do not want to seeking health services?”*

A few participants expressed a lack of trust in hospitals and doctors. They doubted the effectiveness of prescribed treatments, which discouraged them from seeking medical care. This skepticism toward the healthcare system was a barrier to engaging in health seeking behavior.

*I feel that doctors nowadays always require patients to undergo many tests such as CT, MRI, electrocardiogram,* etc.*, and there is no general practitioner who truly relies on experience and medical skills to treat diseases. Another issue is that different doctors have different views on medication. I think many doctors lack skills and clinical experience. (No.8).*

Participants reported that worry about their physical condition and fear of receiving a severe diagnosis contributed to their unwillingness to seek medical care. They worried about the psychological burden and pressure that might result from a diagnosis.

*At first, my eyes were uncomfortable, but I did not dare to go to the hospital to see a doctor. I was afraid the doctor would tell me that my condition was very serious. Later, I had bleeding in the fundus of my eyes, which caused me a lot of psychological pressure. (No. 19).*

Concerns about the risk of infection or transmission in a hospital environment deterred some participants from seeking healthcare. They preferred to avoid hospitals to protect themselves from potential infections.

*I think there are a lot of bacteria and viruses in the hospital, and there are also many infectious diseases. I try not to come to the hospital as much as possible, which is also a form of self-protection. (No.14).*

Some participants expressed reluctance to seek healthcare because they did not want to add financial or care burdens to their families. They were also concerned about causing worry among family members.

*I can only go to the hospital with the company of my family because of I am getting older, but I am afraid of troubling my child. (No.26).*

The discomfort associated with being hospitalized was mentioned as an obstacle. Participants cited issues like poor sleep quality and discomfort in hospital settings as reasons for their reluctance to seek medical care.

*Being hospitalized will also makes me very uncomfortable because I cannot sleep well while someone snores. (No.9).*

### Perception of behavioral cues

#### External clues

*Question: “Are you influenced by external factors when you seeking health?”*

A lot of participates expressed that their belief in health seeking behavior is mainly influenced by their surrounding people, including family, other patients, friends and health care providers (HCPs). The participants feel a strong motivation to seek health services from their family, friends, and health care providers (HCPs), and learn from the indirect experiences of others to influence their own health seeking behavior.

*My friend with diabetes who is drinking while taking medicine has no physical problems, so I should also be OK. (No. 11).*

National healthcare policies had a significant impact on their decision-making.

*The New Rural Cooperative Medical Insurance we purchased has a reimbursement rate of 60% for hospitalization, which is not high and cannot be reimbursed for outpatient services, so I did not easily go to seek health services. (No. 26).*

Having a familiar doctor or a satisfactory health seeking behavior experience are also considered important external factors in promoting patients’ medical treatment.

*The reason why I always go to the People’s Hospital to see a doctor is because I am familiar with the doctors there. They have a better understanding of my condition and know what medication to prescribe. After receiving an infusion, I am relieved. (No. 22).*

#### Internal clues

*Question: “Are you influenced by Internal factors when you seeking health?”*

Participants who had experienced complications due to diabetes were more motivated to seek health services and control their condition, the fear of potential complications drove some participants to seek health services for early prevention.

*Before this cerebral infarction, I did not pay much attention to the management of diabetes. I always felt that the complications were far away from me. After the last cerebral infarction, my mind changed. This time, I went to the hospital immediately when I had symptoms. (No.3).*

Feeling responsible for their family’s well-being encouraged participants to seek health and manage their condition.

*I do not want to seek health services, but when I think about my family, my parents are over 80 years old, and my little grandson still needs my care, so I still have to take care of my body to be responsible for my family. (No. 11).*

Modified health belief model for T2DM patients’ health seeking behavioral decision-making.

**Figure 2:**
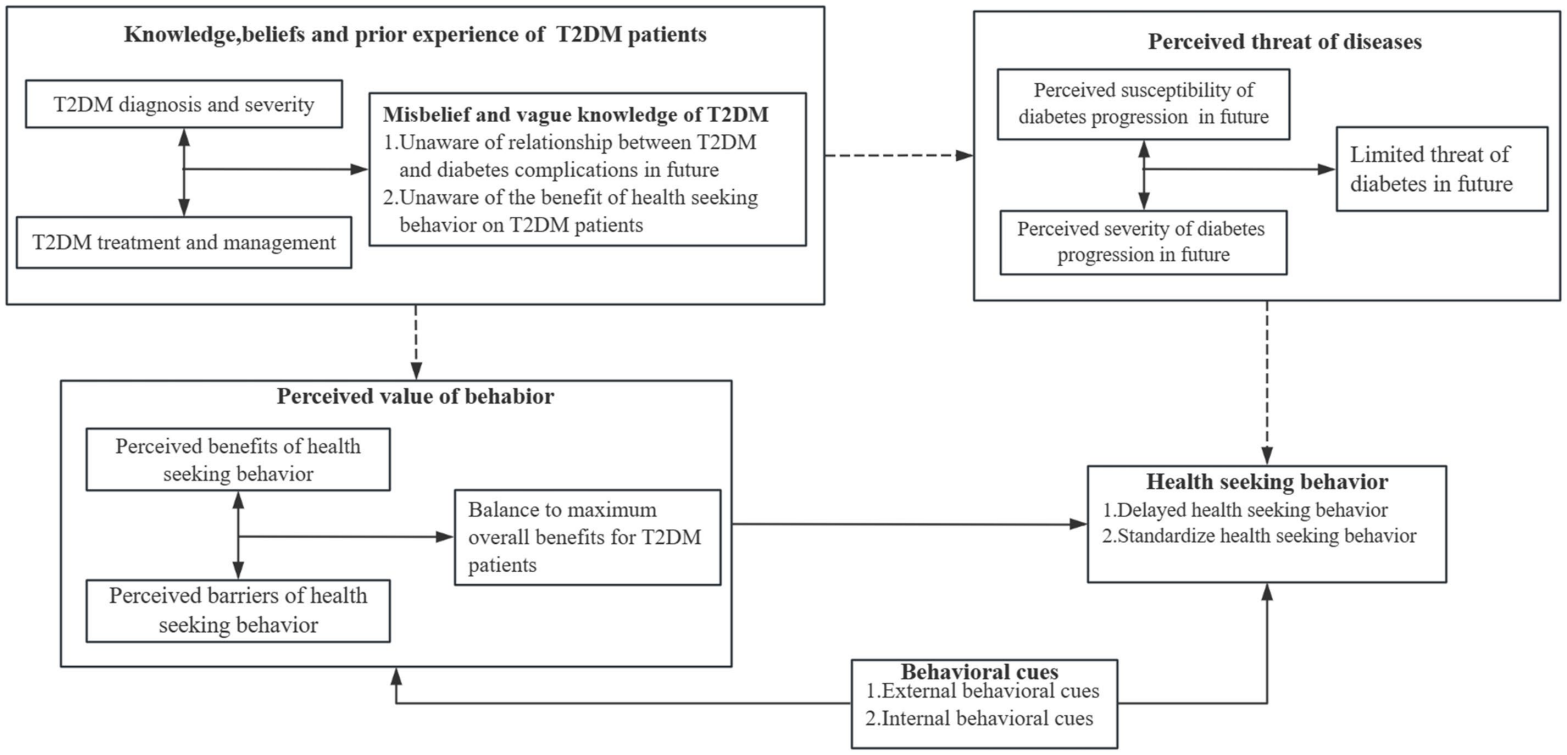
Decision-making process of T2DM patients’ health seeking behavior.

Derived from HBM, Figure 2 presents the decision-making process of T2DM patients HSB. The model showed that T2DM patients may have limited understanding of complications and their long-term impact. Misconceptions and vague knowledge about diabetes deter their awareness of the risk of complications and the benefits of health seeking behavior (Black dashed line in Figure 2). The perception of disease progression risk in T2DM patients plays as a prerequisite role in seeking healthy behavioral decisions. T2DM patients balanced the benefits and barriers of HSB to maximize the overall benefits for themselves that play a decisive role in health seeking behavior decision making. In addition, behavioral cues also facilitate T2DM patients to make reasonable HSB by promoting them to perceive the threat of disease or the benefits of HSB, or by eliminating barriers to medical treatment behaviors. If the perceived benefit of HSB is outweigh than the perceived threat, a T2DM patient is likely to seek health services, while the reverse may be the truth.

## Discussion

### Main findings

A qualitative design has been adopted to explore how T2DM patients developed their health seeking behavior. Based on HBM, the diagnosis and treatment experience of T2DM, their perceived threats of diabetes progression consequence, perceived values of health seeking benefits, behavior clues of health seeking behavior and how these factors influence their health seeking behavior decision-making were combined to form an overall understanding of health seeking behavior decision-making process.

Due to lack of awareness about the disease, T2DM patients often have limited understanding of their condition, leading to underestimating the severity and susceptibility of the diabetes progression, generally considering it as a minor illness that will not significantly impact their lives. This situation prevents T2DM from recognizing the potential health threat of diabetes progression and potential protective effects of HSB, this often results in delayed diagnosis and treatment.

Due to lack of awareness about diabetes, T2DM patients questioned whether diabetes need for medical consultations and tests, leading to underestimating the benefit of the HSB. Financial constraints, time constraints, traffic barriers and other obstacles often results in delayed diagnosis and treatment.

Whether T2DM adopted health seeking behavior depends on the evaluation between the benefit and barriers of HSB for them. External and Internal behavioral cues, such as supports from family and health care providers help T2DM patients reduce the barrier or perceive the benefit, which brought about promotion of HSB.

### Comparison to existing studies

The diagnosis and treatment experience of T2DM patients is considered as the cornerstone of the formation of diabetic patients’ health seeking behavior beliefs (23). T2DM patients typically became aware of their conditions when they experienced symptoms or complications related to diabetes. This also reflects that people generally do not actively pay attention to their own physical health. After diagnosed as diabetes, few people periodic review it, so delayed diagnosis and treatment are common. This is consistent with Jia et al. (12) reported that more than 60% of rural diabetic patients in China have delayed diagnosis and treatment. T2DM patients’ sub-optimal understanding of the disease and poor understanding of HSB were prevalent worldwide, which was also identified in Bangladesh (24), Indonesia (25) and India (26).

Unlike other studies showing that diabetes increases patients’ concerns about disease management and access to health care, and patients struggled to cope with the disease (27), most participants in the current study considered that T2DM was not a severe illness, will not threaten their daily work and life, and their management was arbitrary, just when the condition is severe will one go to seek health services. Unstrict diet control, arbitrary monitoring blood sugar, and even non-indication change of drug use were identified, which was associated with severe adverse events of diabetic patients, such as diabetes foot, diabetes nephropathy and diabetes neuropathy (28). Yang et al. (29) also found a similar phenomenon in a qualitative study. In the current study, young patients with T2DM with a short course of disease (especially those diagnosed as diabetes for less than 5 years), due to the short duration of diabetes, it is relatively easy to control blood sugar in this period. Most of these patients are prone to develop blindly optimistic attitudes in diabetes management, which may reduce their awareness of risks and hinder them from seeking health to avoid potential health threats.

The perceived benefits and perceived barriers to HSB of T2DM patients, largely determine the HSB of them. Consistent with many studies, this study also found that T2DM patients generally believed that diabetes was a condition that they could manage on their own, self-medication and lifestyle adjustments as sufficient, and HSB cannot bring more benefits (25, 30). The established fact that diabetes could not be cured also made them underestimate the benefits of HSB (31). Meanwhile, health care providers play a significant role in T2DM patients’ decision-making process regarding HSB as well as in the diabetes management. However, some participants expressed a lack of trust in the prescriptions and recommendations provided by doctor, believing that many doctors lack medical skills and clinical experience. The main reason why doctors are considered untrustworthy is because they believe they are overly reliant on examination. A quantitative study on the doctor-patient relationship in Chinese public hospitals also confirms the existence of distrust and tension between doctors and patients in China (32).

Fear of spending too much money and time is the two main barriers for T2DM patients in seeking health services. For rural patients with T2DM, medical expenses are considered to be the main obstacle. Due to the low income of rural diabetic patients and the high self-payment expenses (because of high reimbursement threshold and low reimbursement ratio of the new rural cooperative medical insurance), leading to rural T2DM patients usually at a severe stage in their diabetes by the time they receive a treatment. A study in Bangladesh (24) also shows that the lack of medical expenses is the main reason that hinders rural diabetic patients from accessing health services. Besides, inconvenient transportation, the belief that the medical process is troublesome, the fear of being diagnosed with a serious disease, and the fear of adding trouble to the family have also caused huge obstacles to the health seeking behavior of patients with type 2 diabetes. For urban patients with T2DM, the inability to spare time for health seeking was expressed by most participants as the main obstacle. Participants expressed that they were too busy with their work or taking care of their families to take care of their own health, but at the same time, some participants also said that the lack of perception of the seriousness of diabetes progression was the main reason why they used the excuse of no time to rationalize their delayed medical treatment.

On the other hand, tips and support from family members, friends, other patients and healthcare providers are particularly important for T2DM patients’ health seeking behavioral decision-making. T2DM patients feel strong health seeking motivation from them, and learn indirect experience to influence their own HSB beliefs. According to current research, behavioral cues may facilitate the HSB of diabetic patients by reducing the barriers of seeking health services, consistent with the previous studies in other developing areas (33). However, unqualified behavioral cues sometimes cause additional obstacles. For example, in current study, the poor HSB of nearby may hinder them from seeking health services.

### Implication for clinical practices and policy

This study highlighted several factors that should be targeted when formulating intervention measures, and suggested that a multi-facets intervention for health care providers, T2DM patients and their support networks was more likely to promote standardized HSB for diabetic patients.

Firstly, current research has shown that there are disagreements between doctors and patients regarding the examination items, and patients cannot understand the purpose and role of the examination, resulting in a lack of trust in doctors. Encourage joint decision-making between HCPs and patients to enhance trust and understanding can solve this problem. The effectiveness of joint decision-making between doctors and patients in improving the disease perception level of patients with T2DM has been widely confirmed (34, 35). The implementation of doctor-patient joint decision-making is to bring the individual preferences of patients into the decision-making process, which can not only improve patients’ enthusiasm for mastering disease knowledge, but also leading to better compliance and improved the level of health seeking behavior decision-making.

Secondly, HCPs should provide consistent education on diabetes management. Research from the United Kingdom (36) and Sweden (37) shows that experiential education on complications can significantly increase the level of health seeking behavior decision-making by improving the perception of disease threat, and increase T2DM patients likelihood to seeking health services. Due to the fact that HCPs are authorities for public health behavior, both doctors and nurses may play a role.

### Strength and limitations

This study is among the first to use a qualitative approach to explore the development of health seeking behavior in T2DM patients. It provides valuable insights into how patients perceive their condition and make decisions about seeking healthcare. The modified Health Belief Model helps clarify the factors influencing patient behavior. However, the current study also has limitations. The study sample is relatively small, and participants were recruited from specific regions in China, which may limit the generalizability of the findings. There is also the potential for social desirability bias, as participants might provide responses, they think researchers want to hear. Similar studies can be conducted in other countries and cities in the future to explore the different characteristics of health seeking behavior in different cultural backgrounds. Meanwhile we suggested that research on the mechanism of diabetes patients’ health seeking behavior can be carried out in the future to further explore this important theme.

## Conclusion

This study offers a comprehensive understanding of the health-seeking behavior of individuals with T2DM. It explores various aspects related to the diagnosis and treatment experience of T2DM patients and factors that shape their health seeking behavior. Many T2DM patients lack a clear understanding of their condition, often underestimating the severity and susceptibility of the diabetes progression. This situation prevents them from recognizing the potential health threat of diabetes progression and potential protective effects of HSB. The formation of HSB depends on the balance between benefits and barriers of HSB. Therefore, the multi-facets intervention on HCPs, T2DM patients and their networks were of great significance to help T2DM patients form a correct understanding of the disease and standardized health seeking behavior belief.

## Data availability statement

The original contributions presented in the study are included in the article/supplementary material, further inquiries can be directed to the corresponding author.

## Ethics statement

This study received ethical approval from the Ethics Committee of Harbin Medical University (HMUDQ20230418001). Prior to conducting the interviews, participants received both oral explanations and written documentation outlining the study’s procedures, potential risks, and benefits. They provided written informed consent, signifying their willingness to participate in the study and their permission for the interviews to be recorded. To protect participant’s identities and maintain anonymity, their names were replaced with unique identifiers (N01-N26).

## Author contributions

Q-hD: Conceptualization, Data curation, Formal analysis, Investigation, Methodology, Software, Validation, Visualization, Writing – original draft, Writing – review & editing. Z-cZ: Data curation, Formal analysis, Investigation, Methodology, Writing – original draft. YY: Conceptualization, Data curation, Formal analysis, Investigation, Methodology, Writing – original draft. X-xL: Data curation, Formal analysis, Methodology, Supervision, Writing – review & editing. LL: Methodology, Project administration, Resources, Supervision, Validation, Visualization, Writing – review & editing. H-hJ: Conceptualization, Formal analysis, Funding acquisition, Methodology, Project administration, Resources, Supervision, Validation, Visualization, Writing – review & editing.
